# The Medical Current

**Published:** 1887-06

**Authors:** 


### The Medical Current.

Dr. W. E. Reed assumed control of the *Medical Current* last January. The Homœopathic Physician extends cordial greetings to the Doctor in his new field and wishes him the greatest success.

We can emphatically indorse the following platform:

"As a matter of belief and in the interest of which the *Current* will labor, the following principles explain our position:

"I. We believe in the law of *Similia similibus curantur* as the only true and scientific method of treating disease or diseased conditions.

"II. As our remedies are proven singly on the healthy, it follows that the administration of only one medicinal substance at a time is the true and logical deduction from the homœopathic law; and

"III. That if these two principles are carried out the question of the size of the dose becomes a matter easily settled by the individual practitioner."

